# Histone Methyltransferase SETD2 Is Required for Porcine Early Embryonic Development

**DOI:** 10.3390/ani12172226

**Published:** 2022-08-29

**Authors:** Weini Shao, Wei Ning, Chang Liu, Yuanyuan Zou, Yurui Yao, Jiaxin Kang, Zubing Cao

**Affiliations:** Anhui Province Key Laboratory of Local Livestock and Poultry, Genetical Resource Conservation and Breeding, College of Animal Science and Technology, Anhui Agricultural University, Hefei 230036, China

**Keywords:** SETD2, H3K36me3, pig, blastocyst, lineage differentiation

## Abstract

**Simple Summary:**

Normal early embryonic development is important for ensuring sow fertility. Low quality of in vitro production embryos severely limits extensive application of porcine embryo engineering technologies in animal agriculture and the biomedicine field. Histone H3K36 methyltransferase SETD2 reportedly regulates oocyte maturation and preimplantation embryonic development in mice. However, the specific substrate and function of SETD2 in porcine early embryonic development remains unclear. Here, we show that SETD2 preferentially catalyzes H3K36me3 in porcine early embryos. *SETD2* knockdown severely impeded blastocyst cavitation and perturbed normal allocation of inner cell mass and trophectoderm. *SETD2* knockdown caused the apoptosis of cells within blastocysts. Therefore, SETD2 is essential for porcine early embryonic development. These findings provide a better understanding of porcine early embryonic development and lay a potential basis for improving the quality of porcine in vitro production embryos.

**Abstract:**

SET domain-containing 2 (SETD2) is a methyltransferase that can catalyze the di- and tri-methylation of lysine 36 on histone H3 (H3K36me2/me3). SETD2 frequently mediates H3K36me3 modification to regulate both oocyte maturation and preimplantation embryonic development in mice. However, the specific substrate and function of SETD2 in porcine early embryonic development are still unclear. In this study, SETD2 preferentially catalyzed H3K36me3 to regulate porcine early embryonic development. SETD2 mRNA is dynamically expressed during early embryonic development. Functional studies using an RNA interference (RNAi) approach revealed that the expression levels of SETD2 mRNA were effectively knocked down by siRNA microinjection. Immunofluorescence analysis indicated that *SETD2* knockdown (KD) did not affect H3K36me2 modification but significantly reduced H3K36me3 levels, suggesting a preferential H3K36me3 recognition of SETD2 in porcine embryos. Furthermore, *SETD2* KD significantly reduced blastocyst rate and disrupted allocation between inner cell mass (ICM) and trophectoderm (TE) lineage. The expression levels of key genes important for specification of the first two lineages apparently decreased in *SETD2* KD blastocysts. *SETD2* KD markedly increased the apoptotic percentage of cells within embryos and altered the expression of pro- and anti-apoptotic genes. Therefore, our data indicate that SETD2 is essential for porcine early embryonic development.

## 1. Introduction

Epigenetic modifications of chromatin need to be removed soon after fertilization and re-established during blastocyst formation [1,2]. The remodeling of chromatin modifications safeguards the precise spatio-temporal gene expression during early embryogenesis. Previous studies have shown that removal of parentally inherited epigenetic marks except for imprinted genes promotes embryonic genome activation (EGA) and supports the continued development of both naturally fertilized and cloned embryos [3,4]. In addition, the generation of both ICM and TE lineages during blastocyst formation coincides with the re-establishment of epigenetic marks [2,5], implying an involvement of epigenetic modifications in the first lineage specification. Indeed, methylation of the *Elf5* promoter facilitates the specification of pluripotent lineage in mouse embryos [6]. Dimethylation on arginine 26 of histone H3 (H3R26me2) plays an instructive role in the ICM lineage formation [7]. However, the roles of histone lysine methylations in early embryo development are not largely explored.

Histone H3 lysine 36 residue can be modified in the forms of mono-, di- and trimethylation. It is well recognized that SETD2 is the main methyltransferase for catalyzing H3K36me3 in cells [8,9]. A recent study demonstrated the function of SETD2 in catalyzing H3K36me2 in mouse oocytes [10]. Given the unique consequence of each histone methylation [11], it is necessary to identify the specific histone substrate of SETD2 in a specific cellular context. From a functional standpoint, SETD2 has been implicated in various molecular events, such as transcriptional regulation [12], DNA repair [13], DNA methylation [14], mRNA N6-methyladenosine [15], and pre-mRNA splicing [16]. In zebrafish, SETD2-mediated H3K36me3 is dispensable for early embryonic development [17], while H3K36me3 modification catalyzed by SETD2 takes part in the regulation of germ cell differentiation in Drosophila [18]. In mice, zygotic SETD2-deficient embryos arrested at E10.5–E11.5 [8]. Conditional knockout in the germline showed that maternal or paternal SETD2 regulates oocyte maturation [19] and spermatogenesis [20], respectively. Knockdown of *SETD2* by an RNAi approach also impeded blastocyst formation [21]. In addition, SETD2-catalyzed H3K36me3 participates in lineage specification of stem cells [22,23]. Although the expression of SETD2 protein has been characterized in porcine embryos [24], the role of SETD2 in porcine early embryonic development remains unclear.

In the present study, pig embryos were used to examine the expression of SETD2 during early development. Functional studies using an RNAi approach showed that SETD2 specifically catalyzes H3K36me3 modification in porcine embryos. SETD2 is required for porcine early embryonic development. These findings provide new insights into the role of SETD2 in the preimplantation embryo development.

## 2. Materials and Methods

### 2.1. In Vitro Maturation of Oocytes

Ovaries were collected from a local slaughterhouse. Follicular fluid was aspirated from antral follicles at 3–6 mm in diameter. Cumulus–oocyte complexes (COCs) were selected under a stereomicroscope. Subsequently, COCs were cultured in 4-well plates containing maturation medium for 44 h at 38.5 °C, 5% CO_2_ and saturated humidity. Cumulus cells were removed using 1 mg/mL hyaluronidase following maturation.

### 2.2. Parthenogenetic Activation (PA)

Oocytes at the metaphase of meiosis II (MII) were stimulated using two pulses of direct current (1.56 kV/cm for 80 ms) in activation medium (0.3 M mannitol supplemented with 0.1 mM CaCl_2_, 0.1 mM MgCl_2_ and 0.01% polyvinyl alcohol). Subsequently, the MII oocytes were washed in the porcine zygote medium (PZM-3). Oocytes were then cultured in the chemically assisted activation medium for 4 h. Embryos were then cultured in PZM-3 droplets at 38.5 °C, 5% CO_2_ and 95% air with saturated humidity. 

### 2.3. In Vitro Fertilization (IVF)

MII oocytes were washed in the modified Tris-buffered medium (mTBM) containing 2 mg/mL BSA and 2 mM caffeine. Approximately 15 oocytes were incubated in 50 μL droplets of mTBM for 4 h at 38.5 °C in 5% CO_2_ in air. Semen from two boars was mixed and centrifuged at 1900× *g* for 4 min in DPBS supplemented with 1 mg/mL BSA (pH 7.3). Then, sperm was re-suspended with mTBM and adjusted to a proper concentration. Fifty microliters of the sperm solution was added to the mTBM droplets containing oocytes, with a final concentration of 1 × 10^6^ sperms/mL. Following fertilization for 6 h, putative zygotes were incubated in PZM-3 at 38.5 °C in 5% CO_2_ in air.

### 2.4. Real-Time Quantitative Polymerase Chain Reaction (qPCR)

RNA was isolated from oocytes and embryos using RNeasy Mini Kit (Qiagen, Dusseldorf, Germany, 74104). cDNA was synthesized using QuantiTect Reverse Transcription Kit (Qiagen, Dusseldorf, Germany, 205311). The assembly of PCR was prepared in FastStart SYBR Green Master (Roche, Dusseldorf, Germany, 04673514001) and was run on StepOne Plus (Applied Biosystems, Foster, New York, NY, USA). Each experiment was replicated three times for each gene. *EF1A1* was used as the housekeeping gene. The Cq values were obtained and analyzed using the 2^−ΔΔCt^ method. The primers used in this study are provided in Table S1.

### 2.5. Immunofluorescence 

Samples were fixed using 4% paraformaldehyde (PFA) solution for 15 min. Samples were permeabilized using 1% Triton X-100 for 30 min. Samples were then blocked using 2% BSA for 1 h. Samples were co-incubated with primary antibodies overnight at 4 °C. Following washing, samples were co-incubated with secondary antibodies for 1 h in the dark at 37 °C. After washing, samples were stained using 4, 6-diamidino-2-phenylindole dihydrochloride (DAPI, 1 mg/mL) or propidium iodide (PI, 1 mg/mL) for 10 min and were then loaded onto glass slides. For negative control, samples were incubated in blocking solution, omitting secondary antibodies to validate the specificity of commercially available primary antibodies. Finally, images of samples were captured at room temperature using laser scanning confocal microscopy (Olympus, Tokyo, Japan). Signal intensity of targets in nuclear and cytoplasmic regions was assessed using Image J software. The usage information of antibodies is provided in Table S2.

### 2.6. Microinjection

siRNA species was designed to target three different sites of the porcine *SETD2* coding region (GenePharma, Shanghai, China). The siRNA sequences are provided in Table S3. Microinjection was performed in a T2 (TCM199 with 2% FBS) medium containing 7.5 μg/mL Cytochalasin B. Then, 10 pl siRNA solution (50 μM) was injected into the cytoplasm of oocytes. Oocytes were injected with SETD2 siRNA or negative control (NC) siRNA. NC siRNA is an injected control with the injection of sham water. Uninjected oocytes served as the control group.

### 2.7. TdT (Terminal Deoxynucleotidyl Transferase)-Mediated dUDP Nick-End (TUNEL) Staining

Blastocysts at day 7 were fixed in 4% PFA for 15 min at RT. After washing, embryos were permeabilized using 1% Triton X-100 for 30 min at RT and incubated in DNase I (TIANGEN, RT411, Beijing, China) for 1 h at 38.5 °C. After washing, embryos treated with DNase I and enzyme solution without the treatment of label solution (In Situ Cell Death Detection Kit, 7791-13-1, Roche, Dusseldorf, Germany) served as the positive control group. Embryos only treated with DNase I without the treatment of enzyme and label solution served as the negative control group. Embryos without the DNase I treatment in the other groups were directly incubated in enzyme solution for 1 h. Embryos in all groups were stained with DAPI (1 mg/mL) for 15 min and were then imaged using laser scanning confocal microscopy (Olympus, Tokyo, Japan). Exposure time for each channel was consistent among different groups. Total numbers of cells and the numbers of apoptotic dead cells were counted, and the apoptotic ratio was calculated by dividing the number of dead cells by the total number of cells, which included dead cells.

### 2.8. Statistical Analysis

Data were statistically analyzed using one-way ANOVA (SPSS 17.0). Arc-sine transformation was performed for the percentage data. Data were presented as mean ± standard error of mean (mean ± S.E.M). *p* < 0.05 was considered to be statistically significant.

## 3. Results

### 3.1. Dynamic Expression of SETD2 mRNA during Early Embryonic Development

**Figure 1:**
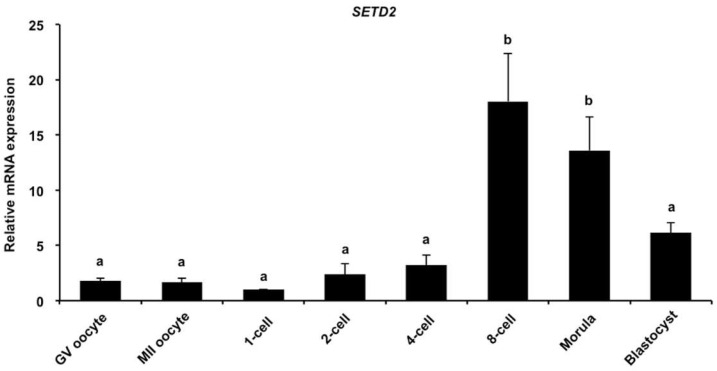
Expression of *STED2* transcripts in oocytes and embryos. Relative expression of *SETD2* transcripts was analyzed by qPCR. The independent experiment was carried out for three times and 40 embryos in each stage were at least included. GV, germinal vesicle; MII, metaphase II. Data are shown as mean ± S.E.M and different letters on the bars indicate significant differences (*p* < 0.05).

To examine the expression of *SETD2* transcripts, qPCR was carried out to analyze the abundance of *STED2* transcripts. We found that the expression levels of *SETD2* mRNA during the development of GV oocyte to the four-cell stage were similar to those in blastocysts, but its expression levels were apparently higher in eight-cell and morula embryos and morula stages than those in oocytes or embryos at the other stages (Figure 1A) (*p* < 0.05). Therefore, SETD2 mRNA was shown to be expressed in porcine early embryos.

### 3.2. RNAi-Mediated Efficient Knockdown of SETD2 mRNA in Early Embryos

**Figure 2:**
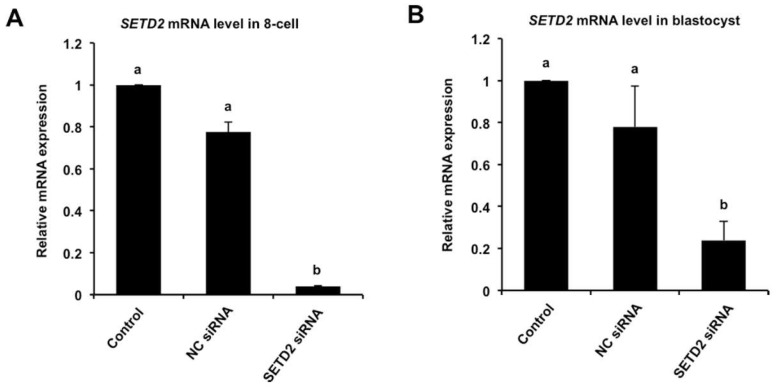
Effect of siRNA injection on the expression levels of SETD2 mRNA. Expression levels of *SETD2* transcripts in eight-cell (**A**) and blastocyst (**B**) among different groups was analyzed by qPCR. The independent experiment was carried out for three times and 30 embryos in each group were at least included. NC, negative control. Data are expressed as mean ± S.E.M and different letters on the bars indicate significant differences (*p* < 0.05).

To investigate the role of SETD2 in early embryonic development, RNAi was performed to delete the *SETD2* transcripts. The data showed that siRNA injection significantly decreased the levels of *SETD2* transcript in eight-cell embryos (Figure 2A) and blastocyst (Figure 2B) compared to the control groups (*p* < 0.05). There were no differences in expression levels between NC siRNA and uninjected control groups (Figure 2A,B). Together, these results document that siRNA injection can reduce *SETD2* mRNA expression in porcine embryos.

### 3.3. SETD2 Knockdown Efficiently Reduces H3K36me3 Levels in Early Embryos

**Figure 3:**
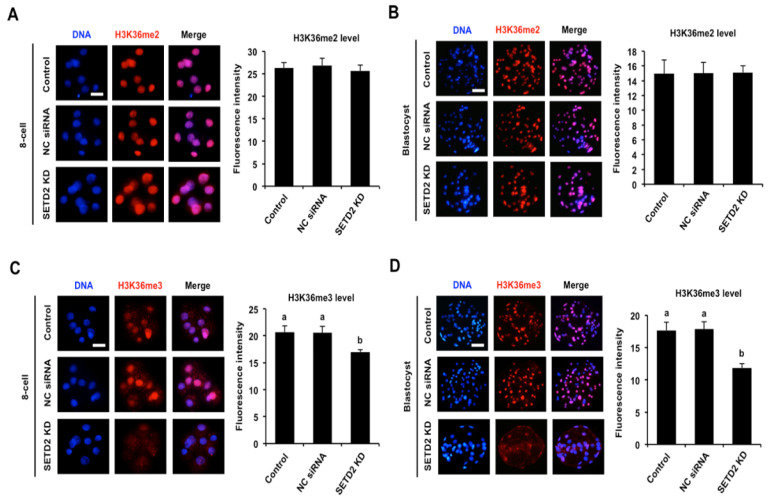
Effects of *SETD2* KD on H3K36me2 and H3K36me3 levels. Immunostaining of H3K36me2 or H3K36me3 in eight-cell embryos (**A**,**C**) and blastocysts (**B**,**D**). Embryos were stained for H3K36me2 or H3K36me3 (red) and DNA (blue). The independent experiment was carried out for three times and 30 embryos in each group were at least included. Representative images obtained by confocal microscopy are shown. Scale bar: 50 µm. Quantitative analysis of fluorescence intensity of H3K36me2 and H3K36me3 is provided in the right panel. The experiment was independently repeated three times with at least 30 embryos per group. All data are shown as mean ± S.E.M and different letters on the bars indicate significant differences (*p* < 0.05).

To ascertain the effects of *SETD2* KD on the levels of H3K36me2 and H3K36me3, immunofluorescence was carried out in early embryos. Fluorescence intensity analysis revealed that *SETD2* KD had no effects on H3K36me2 levels in eight-cell embryos and blastocysts compared to the control groups (Figure 3A,B). As shown in Figure 3C,D, there were no significant differences in the H3K36me3 levels in eight-cell embryos and blastocysts between NC injection and control group. However, *SETD2* KD significantly decreased H3K36me3 levels in eight-cell embryos (Figure 3C) and blastocysts (Figure 3D) stage relative to the control groups (*p* < 0.05). Hence, *SETD2* KD efficiently decreased H3K36me3 levels in porcine early embryos.

### 3.4. SETD2 Knockdown Blocks Blastocyst Development and Perturbs Lineage Allocation

**Figure 4:**
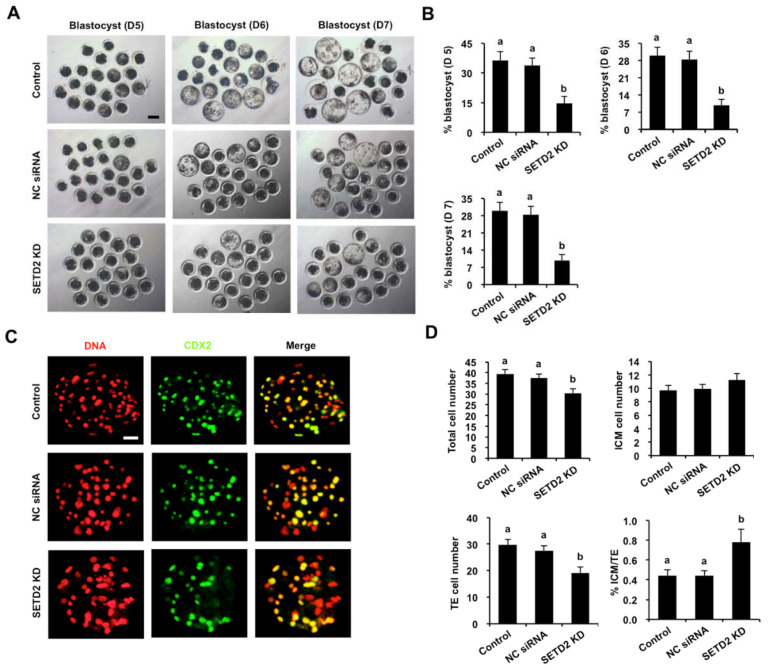
Effects of *SETD2* KD on blastocyst rate and cell allocation. (**A**) Representative brightfield images of blastocysts in each group. Scale bar: 100 µm. (**B**) Developmental rates of blastocysts at day 5, 6 and 7. The independent experiment was carried out three times, and at least 30 embryos in each group were included. (**C**) Representative fluorescence images. Blastocysts in each group were stained for CDX2 (green) and DNA (red). The independent experiment was carried out for three times, and at least 30 embryos in each group were included. Scale bar: 50 µm. (**D**) Cell allocation in embryos from each group. The numbers of total cells, ICM, TE, and the ratio of ICM/TE cells were separately statistically analyzed. ICM: inner cell mass; TE: trophectoderm. All data are expressed as mean ± S.E.M, and different letters on the bars indicate significant differences (*p* < 0.05).

To evaluate the consequences of *SETD2* KD on early embryonic development, the developmental rates of *SETD2* KD embryos were compared to those in NC siRNA and the control group. We observed that *SETD2* KD did not affect the rates of two-cell, four-cell, eight-cell and morula embryos (Figure S1) but significantly reduced the blastocyst rates (Day 5–7) compared to the control groups (Figure 4A,B) (*p* < 0.05). Similarly, *SETD2* KD did not influence the rates of two-cell, four-cell, eight-cell and morula embryos derived from IVF (Figure S2B), while *SETD2* KD significantly reduced blastocyst rates (day 5–7) (Figure S2A,B) (*p* < 0.05). We found no differences in embryo development between NC siRNA injected and the control group (Figure 4A,B and Figure S2A,B). To further determine whether *SETD2* KD influenced lineage allocation, CDX2 staining in embryos was performed to determine the TE cell number (Figure 4C and Figure S2C). The CDX2 negative cells in blastocysts were considered to be ICM cells. We observed that the number of total cells and TE cells was significantly reduced in *SETD2* KD embryos, but the number of ICM cells did not change (Figure 4D and Figure S2D) (*p* < 0.05). Moreover, *SETD2* KD apparently decreased the ratio of ICM/TE cells in blastocysts (Figure 4D and Figure S2D) (*p* < 0.05). Altogether, SETD2 was required for blastocyst formation and the first lineage segregation.

### 3.5. SETD2 Knockdown Perturbs the Expression of Genes Important for the First Cell Differentiation

**Figure 5:**
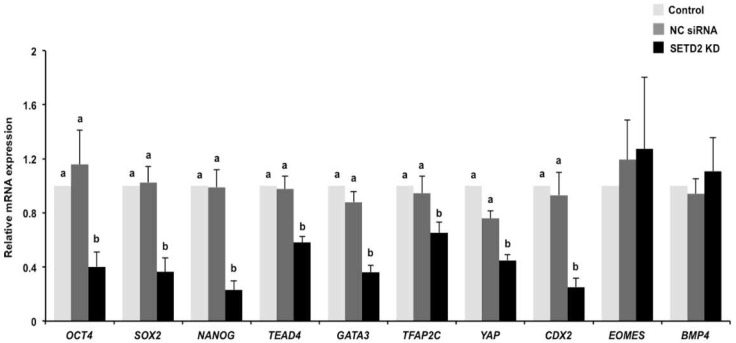
SETD2 KD affected the expression of cell differentiation genes. Expression of genes required for the ICM and TE lineage specification in blastocysts from each group. Expression levels of the indicated genes was analyzed by qPCR. The independent experiment was carried out for three times and 30 embryos in each group were at least included. The data are presented as mean ± S.E.M and different letters indicate significant differences (*p* < 0.05).

To determine the effects of *SETD2* KD on the expression of genes encoding for the markers of ICM and TE lineage specification, the relative mRNA abundance of *OCT4*, *SOX2*, *NANOG*, *TEAD4*, *GATA3*, *TFAP2C*, *YAP*, *CDX2*, *EOMES*, and *BMP4* was quantified by qPCR in day 7 embryos. We found that *SETD2* KD did not affect the expression of *EOMES* and *BMP4* mRNA, whereas it led to a significant reduction in the expression levels of *OCT4*, *SOX2*, *NANOG*, *TEAD4*, *GATA3*, *TFAP2C*, *YAP*, and *CDX2* mRNA (Figure 5) (*p* < 0.05). In addition, the expression levels of the indicated genes in the NC siRNA injection group were similar to those in the control group (Figure 5). Thus, these results show that SETD2 modulates the expression of genes associated with the first cell differentiation in blastocysts. 

### 3.6. SETD2 Knockdown Elevates Apoptosis and Alters the Expression of Pro-Apoptotic and Anti-Apoptotic Genes in Blastocysts

**Figure 6:**
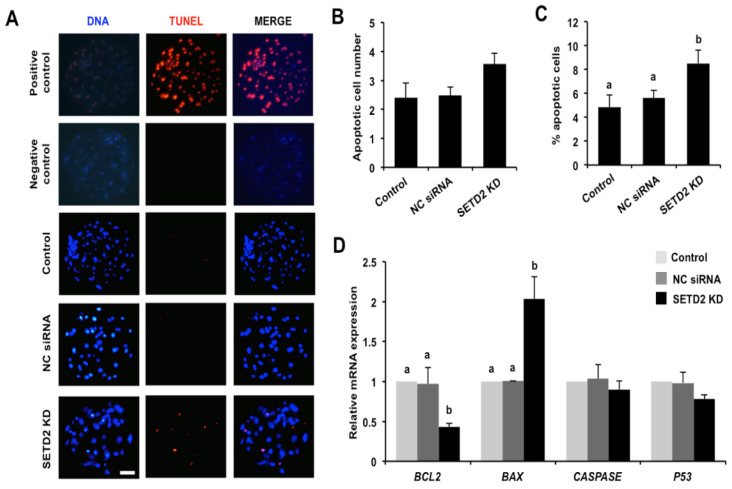
Effects of *SETD2* KD on apoptotic ratio and the expression of pro-apoptotic and anti-apoptotic genes. (**A**) TUNEL staining of blastocysts at day 7. Red spots indicate apoptotic cells, blue spots mark nuclei. Blastocysts treated with DNase I and enzyme solution without the treatment of label solution served as the positive control group. Blastocysts only treated with DNase I without the treatment of enzyme and label solution served as the negative control group. The independent experiment was carried out for three times and 30 embryos in each group were at least included. Representative images obtained by confocal microscopy are shown. Scale bar: 50 µm. (**B**) Analysis of apoptotic cell number in blastocysts. (**C**) Analysis of apoptotic ratio in blastocysts. (**D**) Expression of pro-apoptotic (*BAX*, *CASPASE*, and *P53*) and anti-apoptotic (*BCL2*) genes in blastocysts. Expression levels of the indicated transcripts were analyzed by qPCR. All data are presented as mean ± S.E.M and different letters indicate significant differences (*p* < 0.05).

Considering the protective role of SETD2 in genomic stability prompted us to perform TUNEL staining to evaluate the effects of *SETD2* KD on apoptosis in blastocysts. As shown in Figure 6A, all TUNEL-positive nuclei were observed in the positive control embryos, while there were no TUNEL-positive nuclei in the negative control embryos. Moreover, quantitative analysis showed that *SETD2* KD did not change apoptotic cell number (Figure 6A,B), but remarkably increased the apoptotic ratio of blastocysts (Figure 6C) (*p* < 0.05). Consistent with the increased apoptosis levels, *SETD2* KD not only significantly enhanced expression of pro-apoptotic gene *BAX* but also dramatically reduced the mRNA expression abundance of the anti-apoptotic gene *BCL2* (Figure 6D) (*p* < 0.05). However, *SETD2* KD did not affect the expression of anti-apoptotic genes including *CASPASE* and *P53* (Figure 6D). Hence, these results indicate that SETD2 is important for maintaining genomic stability in porcine blastocysts.

## 4. Discussion

Despite SETD2 regulating both oocyte maturation and pre-implantation embryonic development in mice [21,25], the histone substrate and function of SETD2 in porcine early embryogenesis, especially in the first cell differentiation, are not yet known. We show herein that SETD2 preferentially catalyses H3K36me3, but not H3K36me2 in porcine early embryos. SETD2 regulates blastocyst formation and the first lineage specification. Importantly, SETD2 is also involved in the modulation of genes important for cell differentiation and apoptosis. Therefore, these data demonstrate that SETD2 possibly mediates expression of lineage differentiation and apoptosis genes to regulate porcine early embryonic development.

Recent low-input sequencing of epigenetic marks revealed a critical role of H3K4me3 modification in embryonic genome activation [26]. Both H3K9me3 and H3K27me3 modulated ICM and TE lineage specification during blastocyst development [27]. In a previous study, SETD2 was highly expressed in porcine embryos at the four-cell and blastocyst stage [24], suggesting a possible role of SETD2 in both EGA and blastocyst formation. However, our results indicated that SETD2 is not essential for the development of four-cell to eight-cell embryos, at which stage the EGA occurs in pigs. The finding is aligned with those of previous studies in mice that *SETD2* KD did not impair the development of two-cell embryos [8] and H3K36me3 is uncoupled with EGA [28]. It is worth noting that mouse embryos with depletion of maternal SETD2 arrested at the two-cell stage [19], which suggests a critical role of maternal SETD2 in the EGA. On the other hand, it was observed that *SETD2* KD impaired porcine blastocyst formation, as demonstrated in mouse preimplantation embryonic development [21]. This implies that SETD2′s function in blastocyst development may be conserved between different species.

Histone lysine residue usually presents different methylated statuses and each methylation isoform exerts differential functions in diverse contexts [11]. Although SETD2 is reported to be the primary methyltransfease catalyzing H3K36me3, a recent study revealed that SETD2 is simultaneously implicated in catalyzing H3K36me2 and H3K36me3 in mouse oocytes [10]. In this study, we found that SETD2 preferentially recognizes H3K36me3 but not H3K36me2 in porcine embryos. This suggests that the option of SETD2 for histone substrates likely have a preference between different species or cell types. The developmental asynchrony of early embryos could cause the differential choice for methylation modifications of SETD2 between mice and pigs [29].

The specification of ICM and TE cells is tightly regulated by lineage-restricted expressed transcription factors [30]. The unique expression pattern of lineage determinants is relevant to dynamic changes in histone methylation modifications during early embryogenesis. Indeed, the number of total and TE cells is significantly decreased, whereas the ICM/TE ratio is markedly increased in *STED2* KD pig blastocysts. This indicates that SETD2 regulates the first lineage specification during porcine blastocyst formation. However, we observed that *SETD2* KD did not affect the ICM cell number in porcine blastocysts, which is possibly because SETD2-mediated H3K36me3 is dispensable for the maintenance of pluripotent cells, as shown in mouse embryonic stem cells [31]. Paradoxically, our results indicated that SETD2 regulated the expression of pluripotency genes including *OCT4*, *SOX2*, and *NANOG* in porcine embryos. It is possible that SETD2 promotes the expression of these genes to regulate other embryonic developmental events in pigs. On the other hand, consistent with our results, *Setd2* KD severely disrupted trophoblast lineage differentiation during blastocyst outgrowth in mice [32]. As expected, SETD2 modulated the expression of several TE genes, such as *TEAD4* [33], *GATA3* [34], *TFAP2C* [35], *YAP* [36], and *CDX2* [37]. A number of studies have shown that knockdown or knockout of these genes impairs the TE lineage specification in different species. Thus, we speculate that SETD2 likely mediates the expression of these genes to specify the TE lineage.

Apoptosis is a normal phenomenon for embryos during pre- and post-implantation development under physiological conditions [38]. Excessive apoptosis led to severe defects in the lineage specification, especially in TE differentiation of post-implantation embryos [39]. In this study, we observed that *SETD2* KD increased apoptotic percentage and altered the expression of pro- and anti-apoptosis genes in blastocysts. Thus, the lineage specification defects in *SETD2*-deleted embryos could be due to the excessive apoptosis. High-throughout techniques are needed to determine whether SETD2 directly regulates the expression of lineage specification and apoptosis genes in pigs.

## 5. Conclusions

Our results indicate that SETD2 is required for porcine early embryonic development. Our findings deepen the understanding of the role of SETD2 in the pre-implantation embryo development and broaden the future research on embryo implantation and full-term development.

## Data Availability

The data presented in this study are available on request from the corresponding author.

## Supplementary Materials

The following supporting information can be downloaded at: https://www.mdpi.com/article/10.3390/ani12172226/s1, Figure S1: Effect of *SETD2* knockdown on development of early cleavage-stage parthenogenetic embryos. Figure S2: Effect of *SETD2* knockdown on both development of in vitro fertilization embryos and lineage allocation in blastocysts. Table S1: Porcine-specific primer sequences used in this study. Table S2: Information on primary, secondary antibodies used in this study. Table S3: Information on SETD2 siRNA sequences.
