# Supplementary material for: Histone Methyltransferase SETD2 Is Required for Porcine Early Embryonic Development
Source: Animals (Basel). 2022 Aug 29;12(17):2226. doi: 10.3390/ani12172226 (PMC9454584; doi:10.3390/ani12172226)
Supplement: Supplementary file 1 [file animals-12-02226-s001.zip › animals-1871845-supplementary.pdf]

Figure S1. Effect of SETD2 knockdown on development of early cleavage-stage parthenogenetic embryos

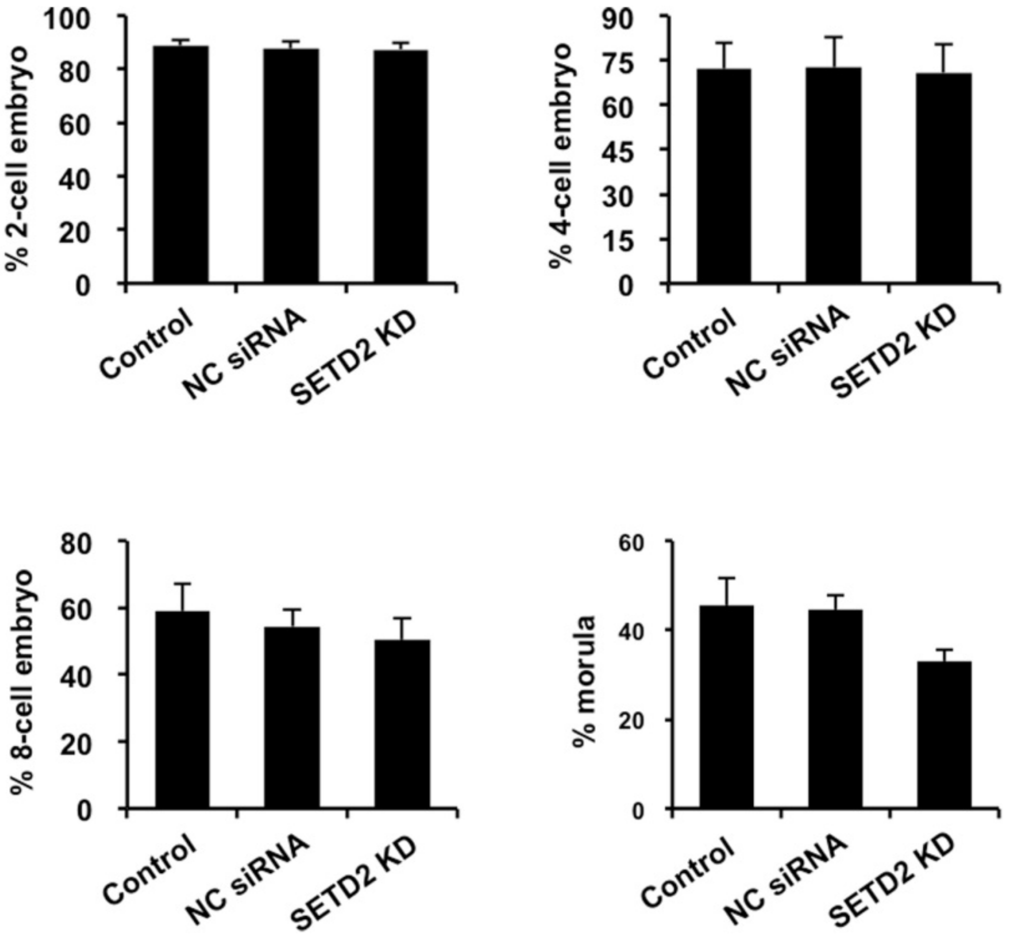

Developmental rates of 2-cell (**A**), 4-cell (**B**), 8-cell (**C**) and morula (**D**) in control, NC siRNA injection and *SETD2* KD groups. All data are shown as mean ± S.E.M and different letters on the bars indicate significant differences ($P < 0.05$).

Figure S2. Effect of SETD2 knockdown on both development of *in vitro* fertilization embryos and lineage allocation in blastocysts

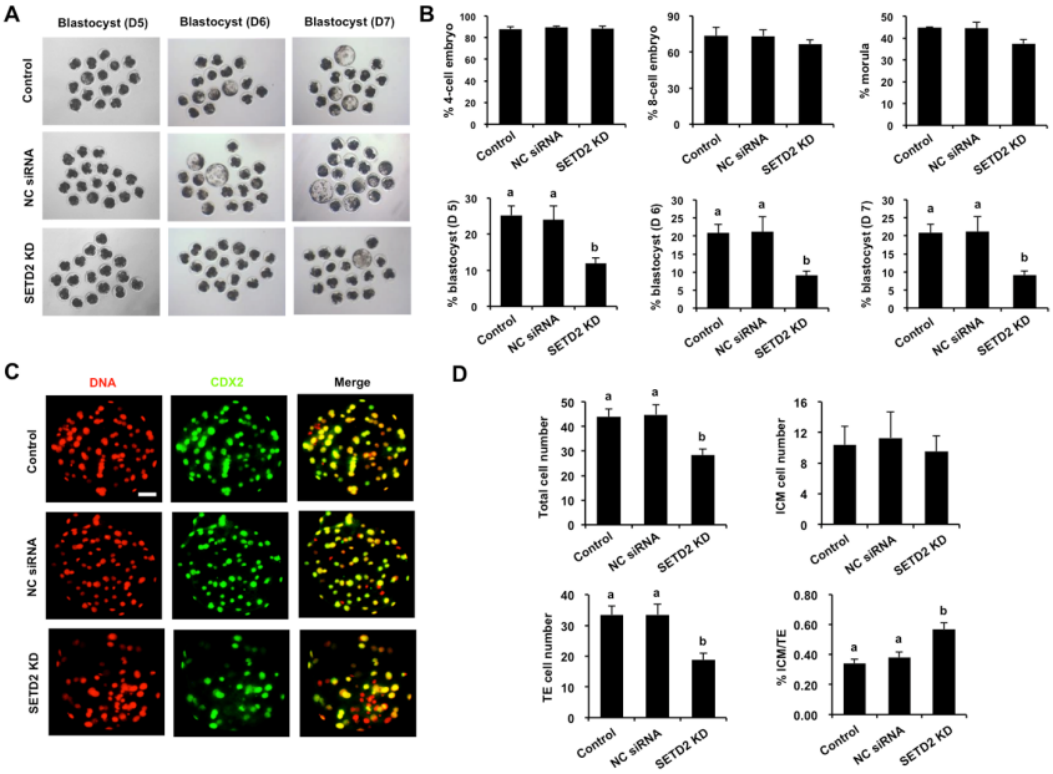

(**A**) Representative images of blastocysts at day 5, 6 and 7 in each group. Scale bar: 100 μm. (**B**) Developmental rates of embryos from the 4-cell to the blastocyst stage. (**C**) Representative fluorescence images of blastocysts. Embryos in each group were stained for CDX2 (green) and DNA (red). The experiment was independently repeated three times with at least 20 embryos per group. Scale bar: 50 μm. (**D**) Analysis of lineage allocation in embryos. The numbers of total cells, ICM cells, TE cells, and the ratio of ICM cells to TE cells were separately recorded and subjected to statistical analysis. ICM: inner cell mass; TE: trophectoderm. All data are presented as mean ± S.E.M and different letters on the bars indicate significant differences ($P < 0.05$).

Table S1. Porcine-specific primer sequences used in this study

Gene symbol	Primer sequences (5'-3')	Product size (bp)	GenBank accession number
SETD2	F: AGCGAATGCAGTGTGAGTGT<br>R: CCCCATTTGGACACCGAGAA	126	XM_021068704.1
OCT4	F: CGAGAACCGAGTGAGAGG<br>R: GGAAAGGAGACCCAGCAG	206	NM_001113060.1
NANOG	F: CTCTCCTCTTCCTTCCTC<br>R: CTTCTGCTTCTTGACTGG	139	NM_001129971.1
TEAD4	F: CATTACTCCTACCGCATCC<br>R: CCTGTGTGTCTCTGTTGG	151	NM_001142666.1
GATA3	F: CACGACACGCTGGAGGAC<br>R: GGCTGGAGTGGCTGAAGG	106	NM_001044567.1
TFAP2C	F: CACGTTCCGGTTGAAAAGGC<br>R: CAGTCCAGGGTTTCGGAGAG	105	NM_001123201.1
YAP	F: AAGCCTCTTGTCTGCTGAGG<br>R: GTTGTAGCGATCCCAGGCTT	118	XM-021062706.1
EOMES	F: CCATCTCCCACGGATTCTCC<br>R: GCACGGTTCTCTCGCCATTA	133	XM_005669315.3
BMP4	F: GGCTGACCACCTCAACTCAA<br>R: CTCAGTTCGGTGGGAACACA	102	NM_001101031
BCL2	F: GGTACCGGAGGGCATTCAGT<br>R: TCCCGGAAGAGTTCGTTCAC	100	NM_214285
BAX	F: TGCTTCAGGGTTTCATCC<br>R: AGACACTCGCTCAACTTC	112	XM_003127290.4
CASPASE3	F: GGATTGAGACGGACAGTG<br>R: CGCCAGGAATAGTAACCAG	109	NM_214131.1
P53	F: CCACCATCCACTACAACTTC<br>R: AAACACGCACCTCAAAGC	135	NM_213824.3
SOX2	F: CGCAGACCTACATGAACG<br>R: TCGGACTTGACCACTGAG	103	NM_001123197.1
CDX2	F: AGTCGCTACATCACCATTCGGAG<br>R: GCTGCTGTTGCTGCAACTTCTTC	139	NM_001278769.1
EF1α1	F: ATTGTTGCTGCTGGTGTTG<br>R: TCATATCTCTTCTGGCTGTAGG	161	NM_001097418-2

F: forward,
R: reverse

Table S2. List of primary antibodies used in this study			
**Primary antibody**	**Species**	**Vendor**	**Cat.no. and dilution**
CDX2	Mouse	Biogenex	AM392 (ready to use)
H3K36me3	Rabbit	abcam	ab9050 (1:100)
H3K36me2	Rabbit	abcam	ab9049 (1:100)
**List of secondary antibodies used in this study**			
**Secondary antibody**	**Species**	**Vendor**	**Cat.no. and dilution**
Alexa Fluor 488 anti-mouse IgG	Goat	Invitrogen	A11029 (1:200)
Alexa Fluor 594 anti-rabbit IgG	Goat	Invitrogen	A11012 (1:200)
HRP-labeled Goat Anti-Rabbit IgG (H+L)	Goat	Beyotime	A0208 (1:4000)

Table S3. Information on *SETD2* siRNA sequences

No. siRNA	Sequence (5'-3')	
	**Sense**	**Antisense**
1	GCACAUACUUCUGAUGAUUTT	AAUCAUCAGAAGUAUGUGCTT
2	CCUUCAGGCUCAGAGUUAATT	UUAACUCUGAGCCUGAAGGTT
3	GCAGUAUUCUCCUGCACAATT	UUGUGCAGGAGAAUACUGCTT
